# The Clinical and Epidemiological Study of Children with Hand, Foot, and Mouth Disease in Hunan, China from 2013 to 2017

**DOI:** 10.1038/s41598-019-48259-1

**Published:** 2019-08-12

**Authors:** Jun Qiu, Haipeng Yan, Nianci Cheng, Xiulan Lu, Xia Hu, Lijuan Liang, Zhenghui Xiao, Lihong Tan

**Affiliations:** 1 House of Journal of Clinical Pediatric Surgery, Hunan Children’s Hospital, University of South China, Changsha, 410007 China; 2 Department of Pediatric Intensive Care Unit (PICU), Hunan Children’s Hospital, University of South China, Changsha, 410007 China

**Keywords:** Viral infection, Epidemiology

## Abstract

Hand, foot, and mouth disease (HFMD) is endemic in the Pacific region, especially in mainland China. The case-fatality ratio of HFMD is increasing steadily. Knowledge of the changing epidemiology of HFMD in different regions is necessary for implementing appropriate intervention strategies. In this study, we describe the clinical and epidemiological characteristics of HFMD in Hunan Children’s Hospital between 2013 and 2017. A total of 7203 patients with HFMD were admitted, with complication and mortality rates of 35.62% and 0.78%, respectively. The total number of children with HFMD, proportion of severely ill children, and HFMD mortality rate were the highest in 2014. The number of cases caused by EV-A71 and CV-A16 decreased continuously, while the number of cases caused by ‘other enteroviruses’ increased yearly since 2014, suggesting that other enteric viruses will gradually replace EV-A71 and CV-A16 as the main pathogenic HFMD agents. Furthermore, EV-A71 and mixed infections accounted for the high case fatality rates in children with severe HFMD, among whom EV-A71 infection resulted in the highest complication and mortality rates; the mild form of the disease was dominated by ‘other enteroviruses’. In conclusion, the changing etiological pattern highlights the need to improve pathogen surveillance and vaccine strategies for HFMD control.

## Introduction

Hand, foot, and mouth disease (HFMD) is a common viral disease in children aged less than 5 years old. It is characterised by fever and rash or herpetic lesions on the hands and feet, as well as exanthema on the oral mucosa and tongue. HFMD may be accompanied by various symptoms, such as bronchitis, bronchiolitis, gastroenteritis, and even neurological manifestations. Children aged 2 years or younger often have more atypical clinical symptoms than older children^1,2^. HFMD is considered a self-limiting disease, and most patients recover within a week; the remainder (a minority) develop meningitis, brainstem encephalitis, neurogenic pulmonary oedema, pulmonary haemorrhage, and circulatory failure within a short time period.

Mainland China is the country most affected by HFMD; it has the highest number of cases and deaths from HFMD. HFMD is commonly seen in male children, and outbreaks occur from May to July^3^. Due to regional differences in geographical location, latitude, climate, and economic status, the intensity, distribution, and trends of the epidemic vary^4^. The World Health Organisation (WHO) reported that, in 2014, approximately 3 million children in China, Japan, Malaysia, North Korea, Singapore, Vietnam, and other western Pacific regions were infected with HFMD, of whom 90% were from mainland China^5^. Recently, sporadic outbreaks have also been described in Europe and North America^6^. In China, the incidence of HFMD disease increased approximately 5-fold from 40/100,000 in 2008 to 198/100,000 population in 2014^7^. Hunan Province had an approximately 6-fold increase in incidence from 55.31/100,000 in 2009 to 318.05/100,000 population in 2014. This indicates that the prevention and treatment of HFMD in China is suboptimal. Therefore, this study aimed to illustrate the clinical and epidemiological characteristics of a population at high risk of HFMD, with a view to provide information for prevention and treatment programmes.

## Results

### Patient characteristics

A total of 7203 patients with HFMD were included in the study; 4580 (63.58%) were boys, and 2623 (36.42%) were girls; the male female ratio was 1.75:1. The median age of all study subjects was 19 months (IQR: 13–29 months).

Among all participants, 96.48% were admitted to the hospital at an early stage of the disease (I and II), and the remaining (3.52%; n = 254) children were admitted to the hospital in stage III or IV of disease. The median duration of hospitalisation for all children was 5 (IQR: 4–6) days. Participants’ characteristics are shown in Table 1.

**Table 1:** Basic characteristics of 7203 children with HFMD.

Variable	Survive(n = 7147)	Death(n = 56)	*Statistics*	*P*-value
Gender(male) n (%)	4540 (99.13)	40 (0.87)	1.42	0.23
Age (month) *M* (*P*_25_~*P*_75_)	18 (14~29)	19 (12~28)	0.58	0.45
Staging^*^				
Phase In (%)	3056 (100.00)	0 (0.00)		
Phase IIn (%)	3887 (99.85)	6 (0.15)	—	<0.001
Phase IIIn (%)	140 (3.33)	10 (6.67)		
Phase IVn (%)	64 (61.54)	40 (38.46)		
complication (%)	2510 (97.82)	56 (2.18)	101.99	0.001
hospitalisation (d) M (P25~P75)	5 (4~6)	2 (0~16.25)	−2.53	<0.001

*Fisher’s exact probability test.

HFMD: hand, foot, and mouth disease.

### Distribution of severity and virus types in different age groups

The case fatality rates of HFMD patients were highest (6113/7203, 84.87%) among children aged less than 3 years old. A minority (12.48%; n = 899) had severe HFMD (SHFMD). The number of SHFMD patients was highest in the 2-year-old group (420/3209, 13.09%) and lowest in the 1–year-old group (153/1617, 9.46%). The proportion of patients with SHFMD in the group aged >2 years was significantly higher than that in the group aged ≤1 year (χ^2^ = 17.92, P < 0.001) (Table 2).

**Table 2:** Distribution of severity of hand, foot, and mouth disease in different age groups.

Age (years)	N	M-HFMD n (%)	S-HFMD n (%)
≤1	1617	1464 (90.54)	153 (9.46)
≤2	3209	2789 (86.91)	420 (13.09)
≤3	1287	1112 (86.40)	175 (13.60)
>3	1090	939 (86.15)	151 (13.85)
Total	7203	6304 (87.52)	899 (12.48)

χ^2^ = 17.92, *P* < 0.001

HFMD: hand, foot, and mouth disease.

The predominant pathogens were ‘other enteroviruses’. EV-A71 and ‘mixed cases of two enteroviruses’ were common in children aged 2 years and older. The predominant pathogen was ‘other enteroviruses’ in children aged ≤1 year (Table 3).

**Table 3:** Distribution of virus types in different age groups.

Age (years)	N	Other enterovirus n (%)	EV-A71 n (%)	CV-A16 n (%)	Mixed infection n (%)
≤1	1617	1177 (72.79)	218 (13.48)	127 (7.85)	95 (5.88)
≤2	3209	1761 (54.88)	680 (21.19)	318 (9.91)	450 (14.02)
≤3	1287	577 (44.83)	269 (20.90)	167 (12.98)	274 (21.29)
>3	1090	412 (37.80)	265 (24.31)	164 (15.05)	249 (22.84)
Total	7203	3927 (54.52)	1432 (19.88)	776 (10.77)	1068 (14.83)

*χ*^2^ = 436.21, *P* < 0.001.

**Figure 1:**
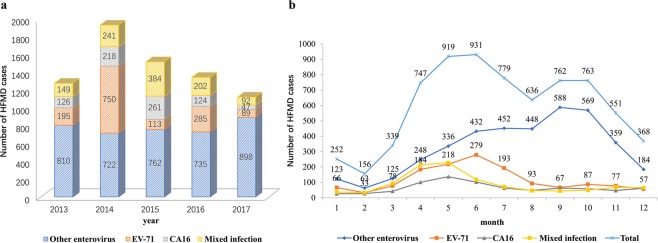
Complication rates and case fatality rates in different age groups.

The overall case fatality rate among children with HFMD was 0.78%. The case fatality rate (20/1278, 1.55%) was highest among participants aged less than 3 years, and significantly higher than that in other age groups (χ^2^ = 14.31, P = 0.003), but was lowest among those aged 3 years and older (3/1090, 0.28%). The complications rate was 35.62% and was significantly higher among those aged >2 years than in those aged ≤2 years (χ^2^ = 164.61, *P* < 0.001) (Fig. 1).

### Virus type trends over time

**Figure 2:**
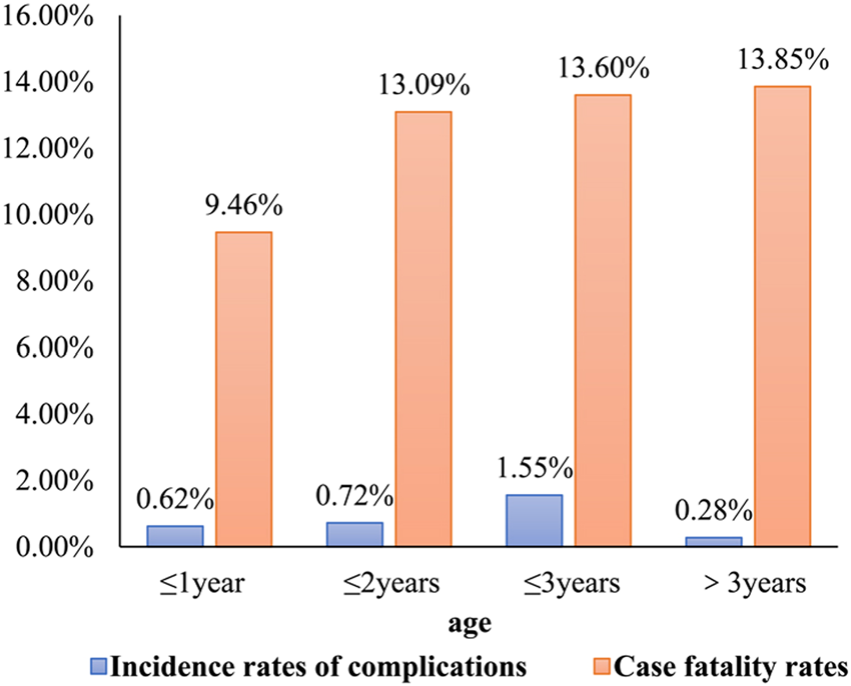
Distribution of virus types in children with hand, foot, and mouth disease from 2013–2017.

The common causative pathogens, EV-A71 and CV-A16, were detected in 1432 (19.88%) and 766 (10.77%) HMFD cases, respectively. EV-A71 contributed the highest number of cases in 2014; whereas the highest number of CV-A16 and ‘mixed infection’ cases were seen in 2015. ‘Other enterovirus’ infections predominated in 2017 (898/1126, 79.75%). There was a statistically significant difference in the distribution of virus types among children over the years (χ^2^ = 1091.74, *P* < 0.001). The proportion of HFMD caused by EV-A71 increased every other year. The positivity rate for CV-A16 was highest in 2015 (261/1520, 17.17%). Cases caused by mixed infections increased from 2013 (149/1280, 11.64%) to 2015 (384/1520, 25.26%) and then decreased until 2017 (92/1126, 8.17%). The positivity rate for other enteroviruses increased from 2014 (722/1931, 37.39%) to 2017 (898/1126, 79.75%) (Fig. 2a).

As shown in Fig. 2b, most HFMD cases were common during the months of May to July and September to October. The positivity rate for EV-A71 reached its peak in June (279/931, 29.97%), while CV-A16 infections peaked in May (137/919, 14.91%).

### Proportion of different pathogens in mild HFMD and severe HFMD

**Figure 3:**
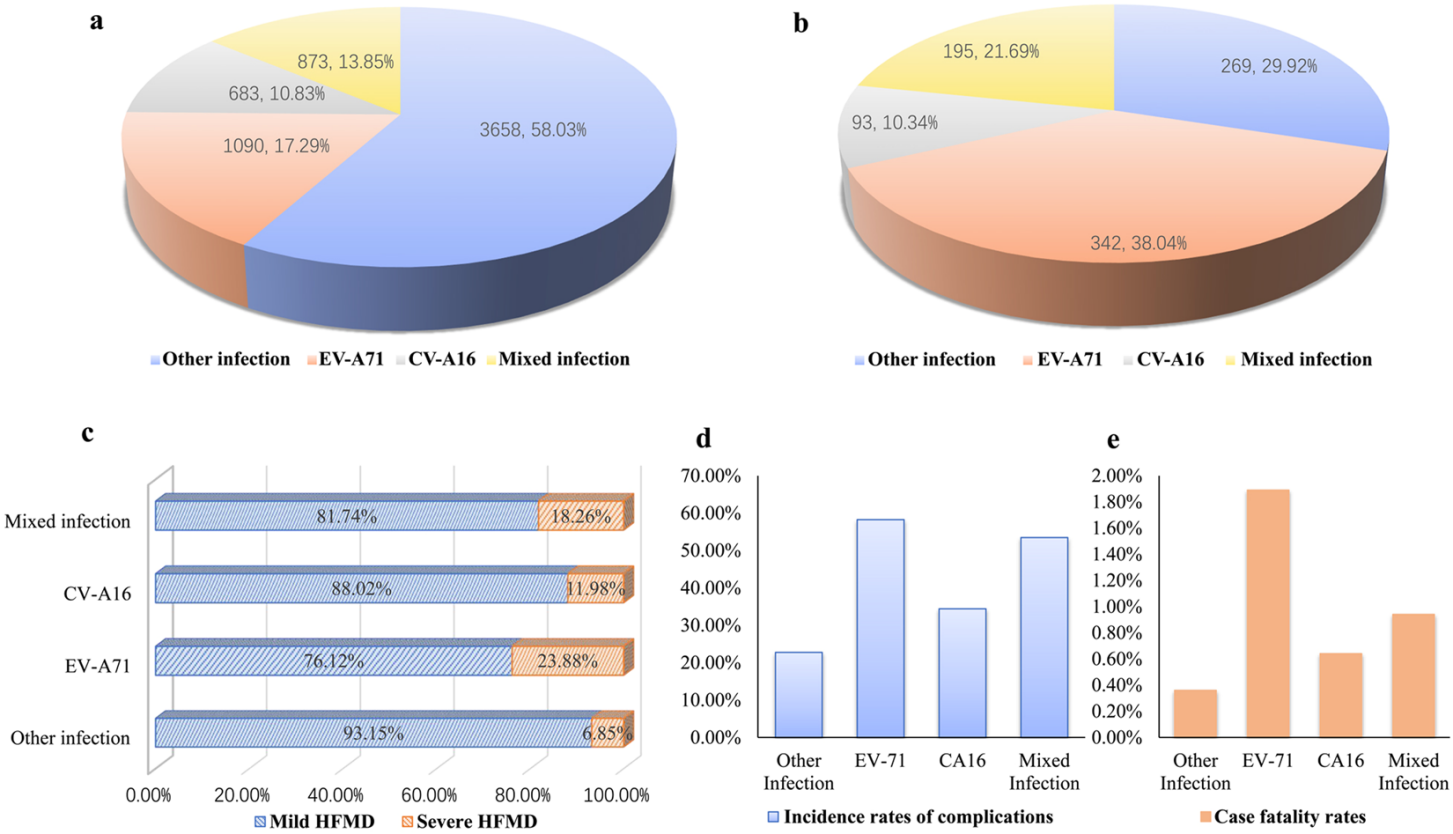
Proportion of different virus types, complication rates, and mortality rates in mild HFMD and severe HFMD. (**a**) Composition of virus types in mild HFMD. (**b**) Composition of virus types in severe HFMD. (**c**) Difference in severity of disease with different virus types. (**d**) Complication rates with different virus types. (**e**) Mortality rates with different virus types. HFMD: hand, foot, and mouth disease.

The pathogens causing mild HFMD were ‘other intestinal viruses’ (58.03%), EV-A71 (17.29%), mixed infection (13.85%), and CV-A16 (10.83%) (Fig. 3a). In severe cases, this was EV-A71 (38.04%), other enteroviruses (29.92%), mixed infections (21.69%), and CV-A16 (10.34%) (Fig. 3b). The etiological agents for the difference in severity between mild and SHFMD, from the highest to the lowest contributors, were EV-A71 (23.88%), mixed infection (18.26%), CV-A16 (11.98%), and other enteroviruses (6.85%). The proportion of SHFMD caused by EV-A71 was higher than that caused by ‘other enterovirus’ (χ^2^ = 317.23, *P* < 0.001) (Fig. 3c).

### Incidence rate of complications and case fatality caused by different pathogens

The incidence of complications caused by EV-A71 and mixed infections were 58.24% (834/1432) and 53.46% (571/1068), respectively. The incidence of complications in children caused by other enteroviruses was the lowest (27.77%; 894/3927), and this was statistically significant (χ^2^ = 751.23, *P* < 0.001) (Fig. 3d). The mortality rate caused by EV-A71 infection was 1.89% (27/1432), which was remarkably higher than that caused by other pathogens. This difference was statistically significant (χ^2^ = 32.34, *P* < 0.001) (Fig. 3e).

## Discussion

The subjects in this study were hospitalised children in Hunan children’s hospital; the only tertiary hospital in Hunan Province with 1,800 beds, including 60 beds in the Infectious Diseases Unit and 80 beds in the Children’s Intensive Care Unit (PICU). Therefore, this study mainly reflects the clinical and epidemiological characteristics of HFMD.

This study illustrated a HFMD case fatality rate of 0.78% among children hospitalised in Hunan Children’s hospital between 2013 and 2017. The main infectious pathogen among 1-year-old children was ‘other enteroviruses’, which had a low complication and mortality rate. The main infectious pathogens for children aged over 2 years were mainly ‘other enteroviruses’ and EV-A71, and mixed infections were also common. The highest case fatality rate was among 3-year-old children. Previous epidemiological data on HFMD indicated that the incidence of SHFMD in China was about 0.9% and the mortality rate was 0.20%-0.39% between 2008 and 2015^8^. South Korean data also showed that the mean age of patients with HFMD was 3.57 years old, and the proportion of severe cases was 1.6%^9^. In our study, 12.48% (899/7203) were severe cases and 56 (0.78%) died. The proportion and case fatality rate of severe cases in this study were higher than the above study. This is because all children included in this study were hospitalised; mild cases that may have been diagnosed at the outpatient clinic were not included. In addition, our study also included severely ill children who were referred from the primary hospital.

Our study also found that susceptible pathogens and case fatality rates varied between ages. This may be due to two main reasons. Firstly, this may be because children under 1 year of age are mainly breastfed, and breastfeeding is a protective factor against HFMD progression^10^. Breast milk may promote the development of the gastrointestinal system of infants and young children which prevents the invasion of foreign viruses. Furthermore, breast milk contains immune substances, immunomodulators, and inflammatory factors, including some antibodies from the mother^11,12^. Secondly, older children partake in more activities and have greater contact with the outside world; therefore, they have more opportunities to contact children with latent infection or infected people.

With respect to time distribution, this study showed that the number of cases, the highest proportion of severe disease, and the case fatality rates were highest in 2014. However, there were no fatalities in 2017. During these five years, the number of HFMD cases increased every other year, and the incidence of complications and mortality between 2014 and 2017 first increased and then decreased. The data from the National Infectious Disease Surveillance Information Management Direct Reporting System between 2008 and 2010^13^ and the data of Fuyang, Anhui from 2008 to 2013^14^ all indicated that outbreaks of HFMD also occur every other year.

In terms of virus type composition, from the results of this study, other enteroviruses may gradually replace EV-A71 and CV-A16 as the main pathogenic causes of HFMD. A survey in Shanghai showed that CV-A6 has replaced EV-A71 and CV-A16 as the main pathogenic cause of HFMD since 2013^15^. Wu *et al*.^11^ conducted pathogen detection tests on 30,377 cases in Hunan Province in 2013 and 2015, and showed that ‘other enteroviruses’ had become the main pathogenic causes of HFMD. These findings support our arguments. These changes may be due to the fact that mixed infections led to genetic recombination of pathogens. In the literature, a patient can have concurrent infection with both EV-A71 and CV-A16 pathogens. This co-infection, which leads to viral gene recombination^12^, could have been responsible for the repeated outbreaks or epidemics of HFMD in China. Moreover, EV-A71 vaccine coverage may have reduced the number of EV-A71 infections. The EV-A71 inactivated vaccine protection rate and antibody production rate are both higher than 90%^16^.

Similar results of changes in virus types were also described in different districts in a Japanese study; CV-A6 was the main pathogen of HFMD in 2011 and the outbreak occurred once every two years^17^. A study in Europe also found a high prevalence of EV-D68 in 14 countries^18^. The outbreak of CV-A6 occurred in different regions of Beijing, Guangzhou, and Shanghai; however, CV-A6 has gradually been replaced with EV-A71 and CV-A16 as the main causes of HFMD in China^19^. A previous report revealed that the EV-A71/CV-A16 combined vaccine also failed to prevent the outbreak of HFMD caused by CV-A10 and CV-A6^20^. Therefore, it is particularly important to develop a multivalent HFMD vaccine to protect children from HFMD caused by enteroviruses^21,22^.

In terms of complications and case fatality rates, our study found that EV-A71 had the highest complication and case fatality rates of 58.24% and 1.89%, respectively. Weng *et al*.^23^ reviewed data from Fujian Province from 2010 to 2014. The proportion of EV-A71 infection in children with the mild form of the disease was 40.23% (5648/14040), that in severely ill children was 72.8% (1194/1640), and in those who died was 98.59% (70/71). Disease surveillance data from Shanghai between 2012 and 2016 showed that 94.05% of cases were mild, and the proportions reported for different pathogens among children were enterovirus (46.88%), CV-A16 (29.63%), EV-A71 (23.28%), and mixed infections (0.21%). From the same surveillance, 5.95% were severe cases and EV-A71 infection (96%) accounted for the majority of infections^24^. According to the national surveillance data, the number of deaths caused by EV-A71, CV-A16, and other enteroviruses in 2008–2015 were 2136, 43, and 129; while the mortality rates were 0.85%, 0.03%, and 0.07%, respectively. This previous mortality rates are similar to the results of this study. Since our study found that EV-A71 infections were concentrated between April and July, we recommend that clinicians should be alert to both EV-A71 and children with severe illnesses during this period in order to implement active intervention measures to reduce the incidence of complications and mortality.

This study had some limitations. The HFMD cases included in this study were drawn from a hospital, and our sample may have been biased. However, the large sample of HFMD hospitalised cases portrays an accurate representation of hospitalised children; a high proportion of our participants had severe disease and provided information that was beneficial for clinical practice. Secondly, all of our participants were drawn from the children’s hospital of Hunan Province; therefore, the population distribution, time distribution, and virus type distribution characteristics of the HFMD patients found in this study are not representative of the whole Hunan Province.

With regards to the composition of virus types, EV-A71 and CV-A16 infections decreased continuously, as the number of cases caused by other enteroviruses increased each year. This suggests that other enteric viruses may gradually replace EV-A71 and CV-A16 as the main pathogenic agents of HFMD. A multivalent HFMD vaccine may be the key to prevent HFMD outbreaks and epidemics in the future. In terms of disease severity, EV-A71 and mixed infections accounted for the high case fatality rates of infections among children with SHFMD, but EV-A71 had the highest incidence of complication and mortality. The mild form of the disease was mainly caused by other enteroviruses.

## Materials and Methods

### Ethical considerations

Permission to conduct this study was granted by the Ethics Committee of Hunan Children’s Hospital (IRB No. HCHLL-2014004). Written informed consent was obtained from the parents and/or legal guardians of each child. All the data were fully anonymised. All experiments were performed in accordance with the approved guidelines and regulations.

### Study subjects

The 7299 hospitalised patients diagnosed with HFMD between the 1^st^ of January 2013 and the 31^st^ of December 2017 in Hunan Children’s Hospital were included in the analysis. Patients were excluded from this study if they had any of the following characteristics: incomplete documentation of information (37 cases, 0.51%); were co-infected with other viruses (10 cases, 0.14%), bacteria (13 cases, 0.18%) or fungi (3 cases, 0.01%); or had concurrent congenital heart disease (17 cases, 0.23%) or primary nephrotic syndrome (6 cases, 0.08%), accompanied by severe fundamental disease or severe immunodeficiency. Finally, 7203 cases were included for clinical analysis.

Data were obtained from the medical records. Information on children with HFMD, including hospitalisation number, gender, date of birth, date of admission, date of discharge, admission to the department, admission stage, admission diagnosis, and complications during hospitalisation (brain encephalitis, pulmonary oedema, pulmonary haemorrhage, circulatory failure), type of virus, mild or severe type, and outcome, was obtained. The diagnostic criteria used were based on the Chinese guidelines for the diagnosis and treatment of SHFMD issued by the Ministry of Health in China^25^.

### Staging of HFMD and definition of related complications

According to the expert agreement on clinical treatment of severe cases of enterovirus 71 (EV-A71) infection HFMD classification criteria, the disease is divided into five phases: eruption stage, neurological stage, early stage of cardiorespiratory failure, cardiopulmonary failure, and convalescence^26^. Evaluation of the severity of disease was performed based on the guidelines for the diagnosis and treatment of SHFMD (2010)^25^. SHFMD was defined as rapidly progressing with meningitis and encephalitis (brainstem encephalitis is the most dangerous), encephalomyelitis, pulmonary oedema, and circulatory disorders which may occur in the first to fifth day of onset. Meningitis, brainstem encephalitis, encephalitis, pulmonary oedema, pulmonary haemorrhage, and circulatory failure are described as follows.

Meningitis: white blood cell counts in cerebrospinal fluid of >10/mm^3^, accompanied by fever, vomiting, headache, irritability, positive symptoms, and signs of meningeal stimulation.

Brainstem encephalitis: frequent spasms, convulsions or acute flaccid paralysis (acute emergence of asymmetrical, non-progressive weakness or paralysis in one or more groups of muscle), reduced or absent deep tendon reflexes.

Encephalitis: children with altered level of consciousness, no febrile seizures, or localised neurological deficit (defined as the presence of non-reflective limb weakness in the acute phase of the child).

Pulmonary oedema: respiratory distress confirmed by chest X-ray.

Pulmonary haemorrhage: alveolar hyperaemia observed on X-ray and dark red foamy sputum or bloody fluid seen through the tracheal tube.

Circulatory failure: children with respiratory distress, tachycardia, pulmonary oedema, and pulmonary haemorrhage.

### EV-A71 and CV-A16 virus detection

Primers were designed based on the species specificity and highly conserved segments of the enterovirus. The EV-A71 VP4-F: 5′-CCGGTGTGCAACAGAGCAAT-3′ and R-5′-TGCGCCACTCGATCACTGTA-3′. The CV-A16 VP1 -F: 5′-GGGAATTTCTTTAGCCGTGC-3′ and R:5′-CCCATCAAR TCAATGT CCC-3′. RNA was extracted according to the experimental procedure provided by the manual, and real-time PCR was performed after reverse transcription. The procedures strictly adhered to the operation standard in the kit. Results were described as ‘Negative defining’: No typical S-type amplification curve or Ct value > 35.1 and ‘Positive defining’: Typical S-type amplification curve with Ct value ≤ 35.1. The reference value was set at 35.1 based on analysis of clinical trial results using the RIC curve method. According to the examination results, children were divided into the EV-71 group, CV-A6 group, and mixed infection group. Children with negative test results but clinically diagnosed HFMD were defined as other enteroviruses groups.

### Statistical analysis

Data entry and statistical analysis was performed using Microsoft Excel 2007 and SPSS 18.0, respectively. The categorical data were presented as frequencies and proportions (%). Categorical variables i.e. the differences in age groups, years, types of enteroviruses, and stage of the disease were compared using Chi-squared (χ^2^) or Fisher’s exact tests. Continuous variables, such as age and length of stay, were summarised as median and inter-quartile ranges (IQR) if the data were not normally distributed. The Wilcoxon signed rank test was used to compare differences in laboratory indicators between the survivor and non-survivor groups. A *P*-value < 0.05 was regarded as statistically significant.

## Data Availability

The dataset generated and analysed in this study are available from the corresponding author on reasonable requests.
